# 

**Published:** 1912-01-13

**Authors:** 


### Florence Nightingale Memorial.

We have received the following contributions since last week. They are for a statue unless otherwise indicated:—

Per Mr. Howard J. Collins: Col. Howard Wilkinson, £2 2s.; Sir John C. Holder, Bart., £1 1s.; Mr. and Mrs. T. H. Russell, £1 1s.; J. H. Lloyd, Esq., £1 1s.; Howard J. Collins, Esq., £1 1s.; "R.S.," 10s. Also from Miss M. J. Phelps, 5s.; Eugene E. Street, Esq., £1 1s. Bristol Centre, per Dr. Watson Williams, in addition to list published December 9 last, "D. and G.," per Miss Collins, 10s.; Mrs. Lucas, 2s. 6d.; Miss Smith, 2s. 6d.

Received by Mr. G. Q. Roberts: The Worshipful Company of Clothworkers, £50; Mrs. Frank Dawes, £10; "B.," £5; Mrs. Taylor, £5; Percivall Currey, Esq., £3 3s.; Fleet Benefit Nursing Association (per Miss Watson, Hon. Sec.), £3 3s.; Lieutenant-Colonel R. Gardiner, £2 2s.; J. C. Harker, Esq., £1 1s.; Miss Hildyard, £1 1s.; Mrs. E. Leighton Hopkins, £1 1s.; Hon. Mrs. Ida Darwin, £1; William Cross, Esq., £1.

### Gifts.

Mr. George Moore, of York, has left £100 to the Doncaster Infirmary.

A bequest of £300 has been left under the will of the late Miss Emma Morrison to the East Sussex Hospital, Hastings.

The Middlesex Hospital has received a donation of £1,000 from Mr. J. D. Bland to endow a bed in memory of his wife.

Mr. William Wright Tasker has bequeathed £500 to the Royal Infirmary, and £500 to the Victoria Hospital for Sick Children in Hull.

Mr. Norton Griffiths, M.P., has sent a donation representing his parliamentary salary for the quarter to the West Bromwich District Hospital.

The London County and Westminster Bank, Ltd., have declared a dividend of $10\frac{5}{8}$ per cent. (less income-tax) for the half-year ended December 31 last, making a total distribution for the year of $21\frac{1}{4}$ per cent.

